# Parkinson’s disease gait rehabilitation at scale: Insights on personalised smartphone-based music cueing

**DOI:** 10.1371/journal.pone.0340106

**Published:** 2026-01-05

**Authors:** Conor Wall, Amber Sacre, Peter McMeekin, Richard Walker, Victoria Hetherington, Yunus Celik, Rodrigo Vitorio, Rosie Morris, Alan Godfrey

**Affiliations:** 1 School of Computer Science, Northumbria University, Newcastle upon Tyne, United Kingdom; 2 Newcastle Patient Safety Research Collaboration, School of Pharmacy, Newcastle University, Newcastle upon Tyne, United Kingdom; 3 School of Healthcare and Nursing Sciences, Northumbria University, Newcastle upon Tyne, United Kingdom; 4 Northumbria Healthcare NHS Foundation Trust, North Shields, Newcastle upon Tyne, United Kingdom; 5 Cumbria, Northumberland Tyne and Wear NHS Foundation Trust, Wolfson Research Centre, Campus for Ageing and Vitality, Newcastle upon Tyne, United Kingdom; 6 Electrical and Electronics Engineering Department, Istanbul University-Cerrahpasa, Istanbul, Turkey; 7 School of Sport, Exercise and Rehabilitation, Northumbria University, Newcastle, United Kingdom; University of Western Ontario, CANADA

## Abstract

This study investigates the perceptions of people with Parkinson’s Disease (PwPD) regarding the use of a smartphone-based music cueing application (app). We present *CuePD*, an app for PwPD to use on their own smartphone for prescribed daily gait retraining/rehabilitation to reduce falls. Patient and public involvement and engagement (PPIE) is fundamental to ensuring the development of *CuePD* as a user-centred platform that effectively incorporates the lived experiences, insights, and needs of PwPD into its design and implementation. A qualitative vignette-based focus group was conducted, comprising seven PwPD and one caregiver, to gather insights on the acceptability and perceived utility of *CuePD*. Through open-ended questioning, participants’ existing routines were explored, including obstacles in sustaining an active lifestyle, and whether *CuePD* could routinely support gait rehabilitation in the home and beyond. The transcript was analysed using a reflexive thematic approach, of which four themes were constructed: (i) exercise preferences and routines, (ii) motivation and engagement, (iii) daily challenges and support, and (iv) app features and usability. Participants expressed a general openness to incorporating *CuePD* into their routines, emphasising the importance of personalised, engaging, and supportive solutions to enhance motivation and adherence to exercise. However, concerns were raised regarding the usability of *CuePD* features and the desire for more feature customisation to meet PD-specific needs and preferences. Overall findings suggest the potential of *CuePD* in supporting personalised and accessible gait rehabilitation through music cueing. *CuePD* may encourage active participation in disease management, better communication with healthcare professionals, and improve the overall quality of life for PwPD. More generally, this study highlights a significant interest among PwPD in using innovative technologies for rehabilitation, pointing towards the need for further development and user-centred design in PD-based mobile health (mHealth) solutions.

## Introduction

Parkinson’s Disease (PD) is a common neurological disorder of which incidence of diagnoses has risen in the past two decades [1]. PD is characterised by its significant debilitating effects on motor functions, including tremors, bradykinesia, rigidity, gait and postural instability [2]. These symptoms can contribute to an increased risk of falling, exacerbated by a heightened fear of falling, and reduction in mobility [3,4]. Subsequently, there can be an increase in the likelihood of future falls, creating a self-perpetuating cycle that can significantly impair daily life [5,6]. Moreover, people with PD (PwPD) often experience a notable decline in their quality of life and find it challenging to maintain independence and an active life within the community [7].

PD symptoms are progressive, necessitating a dynamic and adaptable approach to management and rehabilitation, conforming to the changing needs of the PwPD [8]. This is particularly important as medication can have limited effectiveness in addressing gait and balance symptoms, making physical rehabilitation essential in managing challenges [9]. Typically, the mobility/walking assessment of a PwPD relies on visual gait observation by a clinician or physiotherapist during snapshot appointments in limited spaces which fail to capture real-world challenges. Observations often require the identification of subtle gait disturbances that may contribute to a fall (e.g., erratic step length), but that process is contingent on the observer’s expertise and experience and therefore often lacks standardisation. Accordingly, reliance on visual observation alone is subjective and may mean poor consistency and reproducibility in recognising and identifying gait disturbances within and across PwPD [10]. Nevertheless, subjective visual observations are often used to inform interventions during rehabilitation programmes via gait retraining, such as personalising an external auditory cue, e.g., a metronome, to retrain unstable gait by having the PwPD step to a rhythmical beat to improve stride length and therefore, stability [11].

To better understand gait and more adequately develop a fall intervention and rehabilitation programme, the use of contemporary digital technologies is needed. For example, the use of inertial sensor-based wearables can enable a detailed and objective gait assessment by quantifying high-resolution data across the breadth of the gait cycle [12]. However, most inertial-based wearables for gait assessment are costly and technically complex, limiting their accessibility and ease of use [13]. Moreover, they cannot be used in isolation, with additional technology being required to deliver a cue. There is a need for a holistic and readily accessible but scalable approach for effective gait assessment and intervention delivery to reduce falls where they are most likely to occur, i.e., in the community [14].

Smartphones could provide a pragmatic approach [14,15] as they are ubiquitously carried, while being technology-ready to enable a suite of sensing and communication modalities within the context of gait assessment and intervention delivery [16–18]. For example, smartphones are equipped with inertial sensors and other technologies like speakers, applications/apps, and Internet of Things (IoT)-enabled hardware for remote data capture and transmission [19,20]. This combination has the potential to significantly contribute to the development of tools for reducing fall risk via gait rehabilitation in any setting, enabling a more convenient and accessible alternative to previous approaches. Indeed, the use of ubiquitous technologies (i.e., smartphones, apps) would enable self-management, promoting a greater sense of symptom control for PwPD in their daily lives [21].

Indeed, the involvement of PwPDs in the development of targeted assistive technologies is crucial for ensuring they are acceptable, user-friendly, and unintrusive. Here, we utilise Public and Patient Involvement and Engagement (PPIE) to explore PwPD opinions of a novel personalised music-cueing smartphone app (*CuePD*) for prescribed daily gait retraining/rehabilitation. The technical aspects of *CuePD* have been described elsewhere [22], and some brief details are provided in the Background section for added context. Previously, apps within PD have been suggested for, but strategies for optimal self-management are insufficient [21], as there is little guidance on when they should be used nor considering the needs of the individual. Accordingly, this study aims to understand some lived experiences of PwPD such as (physical) activity habits, daily challenges and perceptions of how a gait retraining app like *CuePD* could be routinely used during their daily lives. The findings from this study can be utilised to improve the app and demonstrate the importance of doing research with, not for, patient groups (https://www.nihr.ac.uk/get-involved/public-involvement). Accordingly, we will capture the nuanced perspectives and experiences of PwPD using the following questions:

1. What are some lived experiences of PwPD regarding their habitual activities?
2. How do PwPD perceive the best use of *CuePD* during their daily lives?

### CuePD: Origins and next steps

The app is described in detail elsewhere including validation, but some rationale for its development and functionality are provided here for context. Firstly, use of a smartphone and app provides a scalable approach to gait rehabilitation/retraining [14]. Different gait retraining approaches have been investigated but auditory cueing is the most effective and practical method [23]. That approach uses rhythmical beats of a metronome to help maintain a steady walking/gait speed [24]. Yet, people listening to metronomes often find it to be monotonous, which can mean a lack of long-term engagement [25,26]. Alternatively, music can be as effective to influence gait while also being more engaging [27–30] but common approaches still lack personalisation, where an individual’s condition and music preferences are not fully considered [31,32]. For example, previous work has focused on non-contemporary musical choices (e.g., classical) that also lack long-term engagement [33].

CuePD was developed to meet the unmet need of a scalable and personalised music-based approach to gait retraining. CuePD harnesses inertial data within a smartphone for near real-time gait assessment and implements an algorithm based on sonification techniques to subtly change the preferred music of the listener to have them step to a new beat/tempo of the music that’s suited to them. In short, the basic functionality and flow of *CuePD* consists of:

1. Assessing a baseline gait cadence (and other gait characteristics) whereby the person walks at their usual pace to determine a personalised cue. Specifically, the baseline cadence/tempo is set to +10% to increase stride length and gait speed while decreasing step time variability based on a previous recommendation [34]. For example, if the baseline cadence is 100 steps/min then the original music track (120 beats per minute, bpm) is adjusted to 110bpm and the person is instructed to walk to that new bpm to encourage 110 steps/min.
2. The listener chooses music from a selection of contemporary playlists, e.g., pop, rock, country with a bpm personalised to them (i.e., While the person is listening to their songs of choice, *CuePD* quantifies stride length, cadence, gait speed and step time variability to objectively determine the cueing effectiveness. Those gait characteristics were chosen based on their usefulness to describe an improvement in gait to determine a reduction in fall risk [34–36].
3. Currently, the functionality of *CuePD* requires the smartphone to be located near the 5^th^ lumbar vertebrae (L5) to align with the functionality of validated algorithms [37–39]. For more details on CuePD such as IoT functionality, please see here [40].

To date, CuePD has been assessed and validated in the lab. Conducting a focus group based on the aims of this study will determine how CuePD should be recommended for use by PwPD in their daily lives.

## Methods

### Study design

This study used a vignette-based focus group to address research questions and was conducted in alignment with the Standards for Reporting Qualitative Research (SRQR) to ensure comprehensive and transparent reporting [41] (S1 Table).

### Sample

Participants were recruited through purposive sampling from a local PwPD support group in the North-East of England. One member of the research team (CW) frequently attended the support groups, where they were able to provide potential participants with a participant information sheet detailing the study particulars, including an overview of *CuePD*. The inclusion criteria were as follows:

- Received a clinical diagnosis of PD or is a PD caregiver.
- Able to self-consent.

Desirable: Experience in mobility-based rehabilitation, including attendance at gym classes for strength training.

### Ethics

Northumbria University Research Ethics Committee approved the study (Ref: 3231, date: 06-03-2023). All participants provided written informed consent before the focus group was conducted and consented to the audio recording of the focus group. The start and end of the recruitment period for this study was 01-04-2023 to 31-03-2024.

### Data collection process

**Fig 1:**
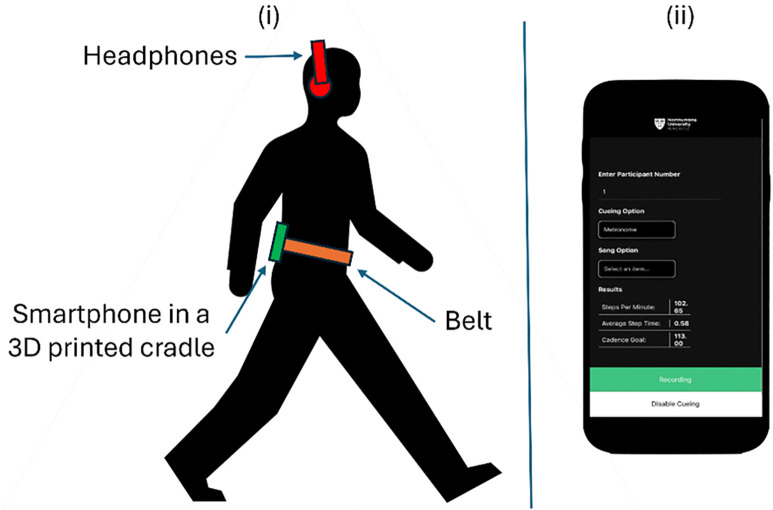
CuePD functionality. (i) Shows how the smartphone device is worn on the body to facilitate current validated algorithm functionality. (ii) Showcases the functionality of the app and what was shown to the participants of the focus group. Icons/images (creative commons licence) from Microsoft PowerPoint were used to populate this figure.

The focus group was conducted in person at Coach Lane Campus, Northumbria University, Newcastle, England. The session featured a semi-structured discussion led by two facilitators (CW, AG) to allow exchanges of views and experiences [42]. A vignette in the form of a video demonstration of how to use CuePD was shown to participants midway through the focus group (see Fig 1 for a screenshot exemplar of the video). The topic guide was informed by PD clinicians (PM and RW) and a qualitative researcher (AS). It was framed around the use of vignettes as “clues” by providing a brief overview of CuePD, allowing participants to fill in the gaps regarding how they would personally utilise the app [43]. Designed to last approximately 90 minutes, the topic guide was as follows: (i) Lived experience of PD (e.g., tell me about your Parkinson’s disease? Tell me about your mobility?); (ii) Daily activity (e.g., what are your preferred methods of exercise?); (iii) CuePD demonstration; (iv) Influences to participate in (physical) activity; (v) Use of other assistive technology (e.g., can you share your experiences of other assistive technology for your Parkinson’s?); (vi) CuePD (e.g., what are your thoughts on using CuePD? What would you change about CuePD?). Relevant prompts and follow-up questions were employed where needed (e.g., can you explain this further?). The focus group was audio recorded and transcribed verbatim (CW), with participants assigned numerical identifiers and other identifiable information removed. A second researcher (AG) validated the anonymised transcript.

### Data analysis

A reflexive thematic approach was used to analyse the transcript [44], a method for identifying, analysing, and reporting patterns or themes within qualitative data. The transcript underwent several coding cycles to ensure the essence of the data was captured [45] (inductive). The codes were then grouped based on unifying sentiments informed by the topic guide (deductive). Thus, thematic analysis was particularly appropriate for this study as it utilised both inductive and deductive reasoning, ensuring the themes provided answers for CuePD development, whilst being grounded within the data. Themes were refined and validated through research team discussions, which exhibited high inter-rater consistency. NVivo software facilitated the analysis process (https://lumivero.com/products/nvivo/).

## Results

**Fig 2:**
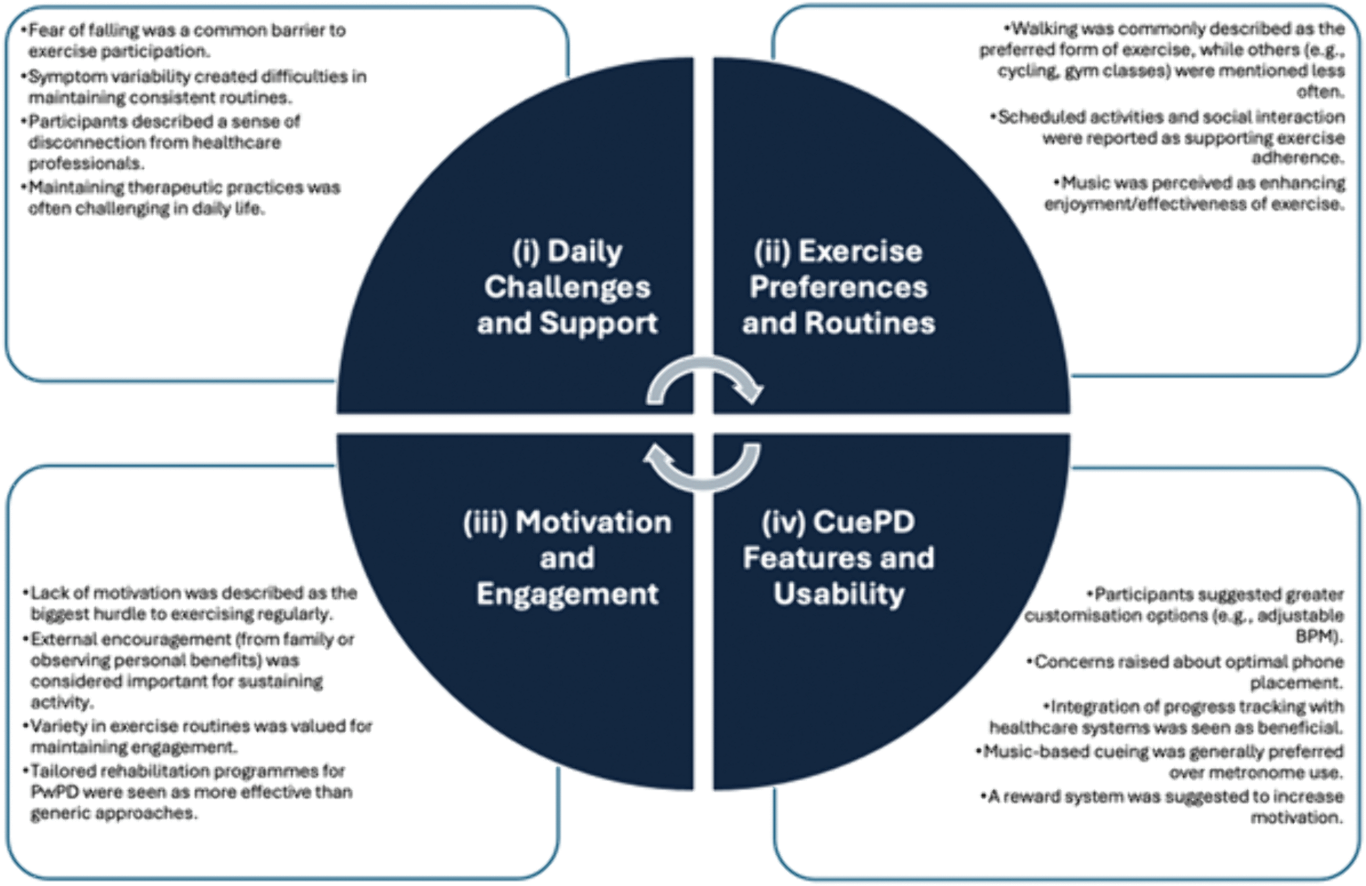
Thematic map, including the themes and codes constructed from reflexive thematic analysis approach of focus group transcript.

Eight participants took part in the focus group, all were 60–73 years of age (3F:4M PwPD, 1M caregiver). The discussion lasted approx. 100 minutes. Four themes were constructed from the reflexive thematic analysis: (i) daily challenges and support, (ii) exercise preferences and routines, (iii) motivation and engagement, and (iv) CuePD features and usability. Fig 2 provides the thematic map, presenting an overview of the themes and their associated codes.

### Theme 1: Daily challenges and support

Theme 1 is contextual, describing some of the gait challenges PwPD face in daily life – namely, symptom variability and fall risk – including some of the ways in which they manage these challenges, such as through contact with healthcare professionals and attending support groups.

Participants described that the challenge of managing the symptoms of PD, such as asymmetrical gait and poor posture, are intensified by their unpredictability; changing from *“day to day, from hour to hour.”* [Participant #1, i.e., P1]

“*…your gait changes throughout the day...sometimes I walk well, and sometimes I don’t. I’m dragging this limb with me…*” [P3]

This symptom variability can make it difficult to schedule exercise.

“*…if you have something [scheduled exercise] that says, ‘right at two o’clock, you’re going to do this’, that doesn’t work. It’s two o’clock, one day, fine. Two o’clock the next day, going to be struggling. So, it’s not something you can sort of say, ‘I’m going to do [at] this time every day’…*” [P8]

A second challenge was the constant fear of falls. One participant suggested that the fear of falling was heightened after their diagnosis, especially regarding “*steps and descending stairs…much more conscious that I might fall*” [P1]. This was met with agreement, with another participant suggesting the fear is increased after a fall has occurred.

*“Yeah, I agree with that. You only have to fall once, and then you’re very aware of that it can happen again”*[P6].

To address these challenges, participants reported engaging with healthcare professionals, PD support groups, and rehabilitation programmes. However, some felt it difficult to often adhere to advice outside of these settings, especially for posture-related issues.

*“P8: When on my own, I fall back into old patterns, like putting my hands in my pockets, then catch myself realising I shouldn’t be doing that. It’s a constant effort to remember to follow the physiotherapists’ advice.*

P3: *For me, it’s the stooped posture. I’m aware it’s getting worse. I find myself constantly looking at the floor.*“

These provide some insight into the importance of continuous engagement and follow-up by healthcare professionals to ensure PwPD maintain the beneficial practices instilled during physical therapy. However, participants voiced a disconnect in their interactions with healthcare professionals, describing a lack of meaningful and/or infrequent engagements in their care.

“*You go to the clinic, you walk up and down, and that’s it. There’s no reflection on what you’re doing at home.”* [P1]

The impact of infrequent contact with healthcare professionals is exacerbated by variability of PD symptoms, as previously discussed.

“*I mean, I see somebody every six months, and it’s just a brief, ‘how have you been?*’*. It’s actually very hard to recall…because you’ve had good days and bad days, bad weeks, and good weeks*” [P2]

This sharp discrepancy underscores a critical gap in care, where the personal needs and day-to-day experiences may largely go unaddressed by those trying to facilitate care.

### Theme 2: Exercise preferences and routines

Theme 2 builds on theme 1, capturing a consensus of how participants liked to exercise and what enhance the exercise experience, such as social interaction and music. There were preferences for accessible and enjoyable forms of exercise; walking was frequently identified by multiple participants as the most favoured activity, with one participant linking this to its simplicity.

*“Walking is definitely the preferred exercise for me.”* [P6]

*“Walking is the easiest.”* [P7]

Reflections illustrate how a low barrier to entry activity (walking) are easily considered in daily life to support well-being. Cycling was another popular form of exercise mentioned by two participants, with one referencing a renewed appreciation for the sport after the COVID-19 pandemic.

*“I got back on the bicycle after 35 years…I find cycling good as well”.* [P1]

The same participant also mentioned they had recently taken up badminton, because it was “*hilarious fun. Nobody cares if you’re bad!*” [P1], suggesting the social aspect of exercise played a significant role in enhancing their exercise experience. This is linked to motivations for exercise, discussed further in Theme 3. Other preferred activities, such boxercise/box-fit and gym-based exercise were also reported. Conversely, participants also discussed their least preferred forms of exercise. The importance of variety in exercise routines was voiced by a participant who critiqued the repetitive nature of some pre-scripted gym workouts.

*“It’s boring. It’s monotonous and repetitive. That’s how gym-based work feels to me.”* [P6]

*“Yeah, the gym is the same.”* [P5]

Another participant felt the same regarding physiotherapy.

*“[Discussing the repetitive nature of exercising in the gym] … so is the physio.”* [P8]

The effectiveness of exercise programs was closely tied to their ability to be tailored to the unique needs of PwPD. Participants critiqued the “*one-size-fits-all*” [P1] mentality often applied to rehabilitation exercise programmes *“among those [physiotherapists] unfamiliar with Parkinson’s…which simply doesn’t work for us”* [P1]. This sentiment was met with agreement.

*“They [physiotherapists] don’t seem to grasp the Parkinson’s aspect. They ask you to perform tasks that are beyond your capability, despite what’s recorded in your history. It’s just not feasible.”* [P7]

Another facet was the impact of music on exercise intensity and enjoyment. Participants remarked on how their favourite music not only helped maintain pace during activities, like walking, but also enriched the overall atmosphere of structured exercise classes.

*“If I’m going for a long walk I pop on my headphones and I think that certainly helps, the music.”* [P6]

*“During physiotherapy and the box-fit* [boxercise] *classes, there’s always music in the background, always music.”* [P4]

The type of music was also important, especially for music cueing.

“*If I listen to like my type of music like Bon Jovi, AC/DC, like type of thing, I’m definitely walking a lot quicker. Compared to if I’m listening to something like Pink Floyd*.” [P8]

Collectively, these narratives provide insight into how varied exercise options, social commitments, PwPD-tailored rehabilitation programmes and music can shape an individual’s engagement and satisfaction with preferences and routines.

### Theme 3: Motivation and engagement

Theme 3 explores motivations for exercising which are both internal (for themselves, the PwPD) and external (for others). Internal motivations were, for example, being able to observe symptom improvement, whereas external motivations were linked to familial encouragement.

Participants stressed the significance of observable benefits as their primary drive for engaging in regular activity and the need to “*commit yourself and keep going*” [P2].

*“For me, the real motivation comes from seeing the difference it makes. When you can see the results, you’re compelled to continue.”* [P3]

*“The fact that it [exercise] works is what makes me want to do it”.* [P1]

The importance of observable benefits was not only limited to exercise, but also to the use of technology, with a participant discussing their use of a different wearable device that provides tactile vibrations to prevent drooling [46].

*“I think it works. It makes a difference and stops me drooling. It motivates me. I’ll do it.”* [P3]

Participants stressed a common need for external encouragement: *“I need to be pushed [in physical therapy]”* [P5]. The call for external impetus was further elaborated by another who observed that effective motivation requires a balance.

*“You need to be pulled as well as pushed.”*[P3]

Familial influence also played a pivotal role in fostering exercise motivation. Participants spoke of the integral support provided by their spouses and children.

*“For me, it’s my wife who initiates our walks, ensuring we stay active.”* [P2]

“*My children are my motivation.”* [P6]

Exercising to raise money for charity was another external source.

*“I’ve just done a charity cycle of 100km. But I did it in tandem with my son.”* [P7]

As discussed in theme 2, some participants favoured scheduled activities, such as badminton and boxercise. The set time and structure also acted as an external motivator, suggesting that having prearranged commitments may be a crucial factor in encouraging participation.

*“Curiously in* [the COVID-19] *lockdown when…you could only go for [limited] hours of exercise a day, that helped me a lot.”* [P1]

*“Committing yourself to a class or badminton session, or whatever, where it’s kind of fixed and you’ve got a teacher involved, or the badminton courts are booked, or whatever. That I think helps, because you kind of have to…”* [P1]

These narratives underscore a multifaceted approach, combining external encouragement, familial support, observable benefits, and personalized strategies with some social structure is essential for motivating and engaging individuals in regular exercise and rehabilitation.

### Theme 4: CuePD features and usability

Theme 4 summarises thoughts on the potential added benefit of *CuePD* for PwPD in their daily lives while highlighting some limitations with its features and usability. Please refer to Methods for an overview of *CuePD*.

Overall, participants found that *CuePD* could be beneficial to their daily lives: *“I think it’s a good idea to me. I think it’s worthwhile. It’s worth trying.”* [P3] Specifically, one participant was impressed with the adaptive feature of *CuePD* that learns from the user.

*“[Discussing how CuePD assesses* an individual’s gait and subsequently delivers a personalised music cue*]…there’s an adaptive bit there, which is very clever.”* [P2]

Participants also stated that they would find the music cueing aspect more engaging compared to the “*monotonous*” [P6] and “*tedious*” [P1] metronome alternative, which some had previously experienced. Also, it was suggested that “*maybe the beat would take over and it would be almost subconscious that I’d be willing to…take the steps…not falling over the curb stones”* [P6].

*“I definitely plod* [walk slowly and heavily] *more when I’m tired. But if I walked with music with a beat, it would definitely help*” [P5].

These perspectives highlight a general preference for the dynamic and more lively nature of music cueing, emphasising the importance of using engaging and stimulating cueing. Additionally, the presence of music is referenced as having the potential to increase confidence when walking and gait speed.

Regarding the occasions when *CuePD* would be best used, participants suggested the app “*would be good for a dedicated exercise period”* [P6], a suggestion echoed by another individual.

*“I would use it walking up and down the street to set my gait. And then go from there.”* [P3]

Others mentioned *CuePD* could be used when completing errands that involve walking.

*“I would be able to use it when I walk to the dentist tomorrow. It would be useful for that half-mile walk, or whatever the distance is. It would also be handy when I’m shopping in stores.”* [P1]

Nonetheless, participants also offered constructive feedback on potential enhancements to *CuePD*, particularly emphasising the need for greater customisation of its features. A key recommendation was to allow users to dynamically adjust the cueing beats per minute rather than it being fixed at +10% above their baseline cadence.

*“I think it might also be useful if you can say, ‘Well, I’m having a bit of a slow day actually, I want to go a bit faster than the 10% that you are now. Or you might say, ‘Actually, I’ve got a sore toe, so actually, I want to do slightly less than that today.’ So being able to put your own choice on it.”* [P1]

A second proposed improvement links to Theme 3, and the idea that observable improvements in PD symptoms can be an internal motivator to exercise. There was an expressed desire for integrated progress-tracking features that could “*record the benefit [of CuePD]*” [P6] and monitor daily performance.

*“What might be useful, is if there’s some way you can record whether you’ve achieved your [goal] gait.”* [P1]

The desire for progress tracking was bolstered by the view that it could enhance the quality of interactions with healthcare professionals by sharing and discussing the data. This is especially relevant when considering how participants felt there was a lack of meaningful and/or infrequent engagements with healthcare professionals.

*“[Describing and example interaction with a healthcare professional] ‘I’ve been using this application, and this is happening…my gaits improving’, or whatever, and ‘I’m now doing exercise regularly’”* [P2].

*“…the healthcare professionals [could] get that information and then they can give the medication accordingly”* [P6].

Despite positive observations, concerns were raised about *CuePD*’s usability and safety. Regarding the usability, participants questioned the practicality of placing the smartphone on the lower back with a need for the phone to remain fully functional for everyday tasks.

“*I think though having it on the bottom of your back, is that not a bit awkward?”*[P8]

*“I think there is a potential issue with wearing the phone on the lower back.”* [P1]

*“…your mobile phone, it’s still got to be your phone. Yeah, you still must be able to do everything else you do on your phone. So, it’s no good at being around your back somewhere. It’s got to be somewhere that you can access.”* [P2]

In view of this, it was suggested that keeping the smartphone in the pocket *“would be best”* [P2].

Safety concerns regarding the use of the headphones were raised, and how this could inhibit participants’ awareness of their surroundings.

*“I’m scared of not concentrating while I’m walking. I’m scared of having a tumble wearing headphones and concentrating on something else.”* [P6]

These narratives reveal a thoughtful and nuanced perspective on *CuePD*, one that appreciates its potential benefits while offering clear guidance for further refinement to better meet users’ needs in real-world contexts.

## Discussion

New approaches are required to develop dynamic treatment strategies in PD that are personalised [47]. Here, the focus was on perceptions of a novel smartphone-based music cueing app, *CuePD,* to retrain gait. The study was guided by two research questions: (1) What are some lived experiences of PwPD regarding their habitual activities? (2) How do PwPD perceive the best use of CuePD during their daily lives? A vignette-based focus group with six PwPD and one care was undertaken, and the transcript was thematically analysed, from which four themes were generated: Theme 1, daily challenges and support; Theme 2, exercise preferences; Theme 3, motivation and engagement; Theme 4, *CuePD* features and usability.

### The lived experience

A key challenge for PwPD highlighted in this study is the fluctuating nature of PD where (physical) impairments can vary from, e.g., hour-to-hour, highlighting the indispensable role of easily adoptable, adjustable exercise activities that can be incorporated readily into daily life. Walking stood out, not only as facilitating routine daily tasks, but as the preferred form of exercise. This is reported elsewhere where the majority of PwPD favoured walking over other forms of exercise [48]. Low participation barriers associated with walking make it an appealing option for PwPD who may find other activities or forms of exercise more challenging or not convenient based on PD fluctuations.

The presence of music during walks (and gym-based exercises) was described to be beneficial, as participants reported that listening to music not only enhances their overall enjoyment of the exercise but can subconsciously encourage them to walk faster. This aligns with existing research, which indicates that music can significantly improve daily exercise engagement by enabling relaxation and reducing anxiety to underlie positive emotional and social outcomes [49]. Indeed, the use of preferred music choice can be extra beneficial to keep users engaged [33,50,51]. Currently, despite its advantages over metronome cueing, the lack of personalisation (own music choice nor aligned with an individual’s gait), contributes to approximately half of PwPD experiencing unchanged or deteriorated gait patterns during a music cueing intervention [52]. As observed, metronome cueing was more tedious compared to the musical alternative, aligning with existing literature [27]. Participants also stressed the motivational impact of perceiving positive outcomes. Experiencing improvements, whether through greater ease in daily tasks or noticing benefits of exercise, reinforced their willingness to remain active [53]. This highlights the importance of integrating mechanisms within CuePD that make such outcomes visible, thereby strengthening engagement over time.

### Perceptions of *CuePD*

Moreover, there was a perception that *CuePD* is a positive innovation worth developing. Specifically, the adaptive personalised cadence (i.e., baseline ability +10%) aspect, was perceived to be advantageous to conform to PD fluctuations and more beneficial compared to other one-sized-fits-all approaches. Furthermore, participants described how their favourite music not only enhanced the enjoyment of exercise but also helped them to sustain activity, e.g., walking at a quicker pace or remaining engaged during longer walks and structured classes. These accounts highlight the importance of aligning cueing with personal music preferences, strengthening the rationale for *CuePD*’s personalised music-based approach compared to generic auditory stimuli.

### Integration challenges

Routine acceptance of *CuePD* is subject to usability challenges. Participants expressed reservations about the location for carrying the smartphone (i.e., lower back). They articulated that this positioning could potentially impede the phone’s accessibility and its usual functionality, with the consensus that integrating *CuePD* usage without disrupting the phone’s primary role is crucial. Participants proposed that relocating the smartphone to a pocket would significantly enhance convenience and ensure the device’s operation remains seamless. That adjustment would better align with the natural use of smartphones, making *CuePD* more user-friendly. Furthermore, the use of headphones and their potential impact on an individual’s fall risk was identified. Additionally, the caregiver perspective highlighted how successful adoption of CuePD depends not only on the willingness of PwPD but also on the support of those around them. Carers/family were seen as crucial in reinforcing use and encouraging ongoing engagement, underscoring their role as enablers of technology-based rehabilitation in everyday life [54].

### Enhancing *CuePD*: Becoming fit for purpose

Current wear location is identified as not practical due to any physical functional limitation for self-placement of the smartphone on L5 and use of an additional attachment. To overcome this, an alternative algorithm is needed. A potential solution could include the collection of simultaneous inertial data from L5 and pocket placements, followed by applying a time-series feature extraction and classification method, such as MiniRocket [55], to examine relationships between both locations. By training a machine learning model on combined sensor data, accurate cadence from pocket inertial data only may be achieved through the transfer of features learned during dual-placement training.

The safety concerns raised about using headphones to listen to cues outdoors could be overcome by using bone conduction headphones. Those headphones have been demonstrated to minimally impact an individual’s situational awareness, providing a safer alternative for such scenarios [56–58]. Additionally, other enhancement features based on focus group discussion should be considered for future work. The Motivation, Engagement and Thriving in User Experience model underscores the significance of supporting basic psychological needs through technology design to increase motivation, engagement, and user wellbeing [59]. That directly aligns with one of the key insights provided during the focus group, which is the critical role of visible and measurable outcomes in cultivating sustained engagement with technology, i.e., PwPD want to see their results. Self-determinant theory emphasizes the relationship between motivation and exercise, highlighting the positive impact of autonomy and competence satisfaction on long-term adherence [60]. That suggests that if *CuePD* produces visible and measurable outcomes it could significantly enhance engagement by satisfying basic psychological needs [61]. A systematic review of the efficacy of app-based interventions for improving activity indicates that technology can play a pivotal role in facilitating regular activity through the provision of real-time feedback and measurable outcomes [62]. Accordingly a future iteration of *CuePD* will include intuitive feedback, and measured outcomes that present information in a clear and engaging manner [63].

The complexity of PD symptoms and differences in technological literacy necessitate personalised approaches, making it critical to engage PwPD to ensure all technology meets their needs, enhancing long-term engagement [64]. While participants recognised the app could be adaptive to the persons baseline gait (aligning with PD fluctuations) they also expressed a desire for a more nuanced personalised approach by enabling them to adjust the app’s +10% functionality based on their physical condition but also their own personal ambitions, i.e., manually adjusting the cadence goal (e.g., 1–10%) depending on their symptoms. However, implementing a functional whereby the PwPD has (daily) control of the cue intensity should be treated with caution as the person may not select a high enough intensity to promote improvements in gait performance. Specifically, PwPD may need to be pushed at higher intensity levels, beyond their voluntary limits, to induce gait performance changes and to evidence app efficacy to reduce falls [65].

### Bridging a gap

Findings suggest there is a critical need for patient-centred tools that enhance communication and foster a more collaborative, well-informed approach to managing PD [66]. This issue is common amongst PwPD, as they often receive insufficient care due to challenges in accessing and coordinating community-based healthcare, inadequate home support for increasing needs, and institutional inflexibility, leading to a lack of continuity, personalisation, and integration of care [67]. The integration of wider PD symptom logging and a communication conjugate to wider health network extends beyond the immediate purpose of this research but is worth noting. Beyond enhancing communication with healthcare professionals, participants also suggested that CuePD could support the optimisation of pharmacological management, discussing its potential utility in further identifying how gait performance can vary with medication timing [68]. This reflects a wider opportunity for solutions, such as CuePD, to provide clinicians with ecologically valid insights into medication efficacy, complementing snapshot assessments in clinical settings and contributing to more personalised treatment strategies in PD.

### Strengths and limitations

This study’s qualitative methodology, utilizing a vignette-based focus group, was pivotal in capturing the nuanced perspectives of users regarding CuePD. The discussion allowed for a sharing of experiences and perceptions through PPIE, allowing PwPD to take an active role in shaping targeted assistive technology. The themes constructed in the analysis provided a detailed account of *CuePD* ‘s integration into users’ daily lives, its utility, and areas for enhancement based on actual user experiences. The reflexive thematic analysis deepened our understanding of how the app could meet the needs of PwPD, highlighting its strengths in offering potential improvements in usability and engagement while also identifying areas for refinement.

The methodology has some limitations such as use of one focus group consisting of a relatively small, homogenous group of participants limits the generalisability of its findings to the broader PD community. Additionally, the participants involved may be more inclined to embrace technology due to their openness to research that incorporates technological tools. As a result, this analysis may not fully capture the wide range of challenges and preferences present among a more diverse population with PD. Furthermore, the discussion indicated a need for ongoing engagement with the target audience to refine the app, suggesting that initial findings may not fully represent the complexities of integrating technology into PD rehabilitation.

Although only one caregiver took part, their contribution provided valuable secondary insight into the shared challenges of symptom management and motivation when supporting a PwPD. However, relying on a single perspective is a limitation, and future research should seek to engage a wider range of caregivers to more fully capture further insights and their role in facilitating both CuePD use and rehabilitation.

## Conclusion

This study provides insights from a focus group into the perceptions of *CuePD* which has the potential to retrain gait in the home and/or community via music underpinned by a personalised feature to adapt to the fluctuating needs of PwPD. Findings reveal a weak preference for scripted activities to manage PD with a stronger preference toward lower barrier activities that were routine within daily life where walking was key. Accordingly, when considering low barrier and routinely integrated approaches, *CuePD* would be best used by PwPD at their discretion during leisurely walks compared to prescribed days and/or time. However, technical limitations of *CuePD* were identified that will need to be overcome to ensure its pragmatic use. This study lays the groundwork for further development and refinement of the app, aiming to better meet the needs of PwPD, enhancing their independence, and improving their quality of life.

## Supporting information

S1 Table Standards for reporting qualitative research (SRQR). (DOCX)
